# Perfectionism and Effort-Related Cardiac Activity: Do Perfectionists Try Harder?

**DOI:** 10.1371/journal.pone.0160340

**Published:** 2016-08-02

**Authors:** Kelly L. Harper, Kari M. Eddington, Paul J. Silvia

**Affiliations:** Department of Psychology, University of North Carolina at Greensboro, Greensboro, North Carolina, United States of America; University of Groningen, NETHERLANDS

## Abstract

Do perfectionists try harder? Previous research on perfectionism and effort has used self-report items and task performance as indicators of effort. The current study investigated whether individual differences in perfectionism predicted effort-related cardiac activity during a mental effort task. Based on past research that suggests adaptive perfectionism is associated with higher effort, it was hypothesized that self-oriented perfectionism (SOP) would predict increased effort on the task. One hundred and eleven college students completed the Multidimensional Perfectionism Scale (MPS) and a self-paced parity task in which they received a small cash reward (3 cents) for each correct response. Impedance cardiography was used to assess autonomic reactivity, and regression models tested whether SOP and socially prescribed perfectionism (SPP) explained autonomic reactivity. Overall, participants showed both sympathetic (faster pre-ejection period; PEP) and parasympathetic activation (elevated high-frequency heart rate variability; HRV) during the task, reflecting higher effort and engagement. Contrary to predictions, individual differences in perfectionism did not moderate cardiac reactivity. These findings draw attention to the importance of assessing physiological components of effort and motivation directly rather than inferring them from task performance or self-reported effort.

## Introduction

Perfectionism is a personality construct characterized by having unrealistic performance standards and excessively critical self-evaluations [1]. Although perfectionism has been measured in different ways, it is widely recognized that perfectionism is a multi-dimensional construct with adaptive and maladaptive dimensions [1–3]. The adaptive form of perfectionism consists of intrinsic and self-directed beliefs for striving for perfection [1], and it is associated with positive domains of well-being such as personal growth [4], adaptive coping [2,5], and high self-efficacy [6]. The maladaptive form of perfectionism, however, consists of extrinsic beliefs for holding perfectionist standards—believing that others have unrealistic and unattainable expectations for them [1]—and it is associated with depression and anxiety [7], hopelessness [8], low self-efficacy [9], and avoidant coping styles [2,5].

In addition to emotional outcomes, maladaptive and adaptive forms of perfectionism relate differently to performance outcomes. People high on adaptive perfectionism tend to achieve higher grades and perform better on academic exams [10–12] and music competitions [12]. In contrast, maladaptive perfectionism doesn’t consistently relate to performance. Some studies have found that maladaptive perfectionism has a negative relationship with achievement [11,13], but other studies have found no relationship [6,14]. Overall, the adaptive component appears consistently related to high achievement, but the maladaptive form is negatively or unrelated to performance.

One reason adaptive perfectionism could affect achievement is that perfectionists may put more effort into achieving their goals. Few studies have directly examined the relation between perfectionism and effort. Two studies found that musicians high on adaptive perfectionism had higher effort, which was measured by time spent practicing a musical instrument [12,15]. In another study, people high on adaptive perfectionism rated higher subjective effort during a lab task [14]. Self-reported time and effort, however, have limitations as indicators of effort. First, time spent on a task and effort are not necessarily related: people might work intensely for brief periods or listlessly for long periods [16,17]. Second, self-report items, while widely used in motivation research, are sensitive to demand characteristics and self-presentational issues [18].

Because of the limitations of behavioral and self-report indicators of effort, a large tradition in biological psychology has developed that assesses physiological activity linked to effort. Research rooted in Brehm’s motivational intensity theory [19,20], for example, emphasizes effort-related cardiovascular changes that occur when people confront both physical and mental challenges [21,22]. A contemporary measure of beta-adrenergic sympathetic activation, the cardiac pre-ejection period (PEP), is often used as a physiological indicator of mental effort. As the sympathetic branch of the autonomic nervous system become more active on the heart, it contracts more forcefully, which is apparent in faster PEP scores [23]. Although sympathetic measures of effort are more common [24], recent research has emphasized examining both branches of the autonomic nervous system. Grit, a personality construct that captures the ability to sustain effort towards long-term goals, was related to co-activation of the sympathetic and parasympathetic branches [25]. Specifically, Perseverance, a subscale from the Short Grit Scale [26], predicted higher levels of effort engagement—reflected in faster PEP and higher RSA—during a mental effort task. This finding draws attention to the importance of looking at both branches of the autonomic nervous system during mental tasks.

To date, only one study has examined perfectionism and cardiac reactivity. Besser, Flett, Hewitt, and Guez [27] looked at how maladaptive and adaptive perfectionism interacted with task performance, confidence, and manipulated feedback in predicting systolic blood pressure (SBP), a measure of sympathetic activation [24]. Although this project showed a relation between perfectionism and sympathetic activation, it did not measure physiological activity during the task. Therefore, its findings reflect effects of perfectionism on reactions to false feedback rather than to the physiological engagement of effort when faced with a challenge.

In addition to examining motivational aspects of perfectionism, using physiological measures in research on perfectionism can help bridge basic scientific research on perfectionism with health outcomes. Specifically, recent research indicates that high levels of perfectionism may increase mortality risk [28]. Moreover, research on perfectionism and coping suggests that perfectionists may have dysfunctional stress responses, which may explain some of the increased risk for maladaptive health outcomes [29]. The use of physiological measures, such as cardiac reactivity, in the perfectionism literature may provide additional information on the biology of perfectionism and help connect the behavioral research on perfectionism to health psychology research.

### The Present Research

The current study examined effort-related physiological activity in people varying in levels of perfectionism during a self-paced task with a cash incentive for each correct response. When people can work at their own pace, effort is proportional to the value of the outcome [30]. In these cases, motivational intensity theory predicts, and many studies have found [31], that the intensity of effort is a function of the importance of the goals and incentives at stake. If perfectionism affects effort, it should increase effort by making achievement and success more important. When confronted with a challenge in which effort is a function of goal importance, people higher in perfectionism should thus exert more effort, reflected in higher sympathetic reactivity from baseline to task.

Perfectionism was defined using Hewitt and Flett’s [1] model, which has three domains of perfectionism: *self-oriented perfectionism* (SOP), *socially prescribed perfectionism* (SPP), and *other-oriented perfectionism* (OOP). Within this model, SOP and SPP are viewed as adaptive and maladaptive, respectively. We examined whether self-oriented perfectionism (SOP) and socially-prescribed perfectionism (SPP) predict decreased effort (indicated by a decrease in sympathetic activation, defined by higher PEP values) or increased effort (an increase in sympathetic activation, defined by lower PEP values) during the task. As a secondary outcome, respiratory sinus arrhythmia (RSA), a common marker of heart rate variability, was measured to examine whether perfectionism predicted changes in parasympathetic activation during the task.

Based on previous research on self-reported effort, we expected that SOP would predict a decrease in PEP values, indicating increased effort on the task. Based on the inconsistent findings to date, no specific predictions were made for SPP and effort. Finally, as a secondary hypothesis, we expected SOP to predict increased RSA reactivity, indicating a co-activation profile [32,33] and increased self-regulatory control [34], similar to the finding for grit and RSA [25].

## Methods

### Participants

Participants (*n* = 111) were adult female undergraduate students who received credit toward a voluntary research option in a psychology class. From the initial sample, 10 participants were excluded because they indicated taking medications that modify cardiac autonomic activity (e.g., psychotropic medication for depression, anxiety, and attention-deficit/hyperactivity disorder); 1 person was excluded because the session was interrupted by a fire alarm; and 2 people were excluded because they didn’t understand the parity task. Only female participants were recruited because the small proportion of men in the research participation pool and the limited availability of male research assistants made recruiting roughly equal numbers of men and women impractical.

The 98 participants that were included in analyses had a mean age of 18.53 (*SD* = 1.06, age range: 18–23 years), and 43.9% were African American, 44.9% were Caucasian, 6.10% were Asian, 7.1% were Hispanic, 1.0% were Native American, and 2.0% declined to state their ethnicity. (Participants were invited to indicate more than one category.) The mean body mass index (BMI) was 24.11 (range: 16.82–46.51). Every participant signed informed consent, and the study was approved and monitored by the UNCG Institutional Review Board.

### Procedure and Materials

Participants took part individually, and all sessions were run by a same-gender (female) experimenter, who explained that the study was about how the body responds during mental tasks and challenges. The experimenter placed the electrodes and allowed a few minutes to pass before recording to ensure the signals were clean and stable. The participants then completed a baseline period, in which they completed demographic surveys and questionnaires (administered using MediaLab; Empirisoft, NY). Values from the last 5 minutes of the baseline period were used for the physiological baseline scores.

#### Assessment of Perfectionism

The Multidimensional Perfectionism Scale (MPS; [1]) was used to measure perfectionism. The MPS is a 45-item scale measuring three dimensions of perfectionism: self-oriented perfectionism (SOP), socially-prescribed perfectionism (SPP), and other-oriented perfectionism. Items capture individual differences in perfectionism within these three domains (e.g. “One of my goals is to be perfect in everything I do”, “I find it difficult to meet others’ expectations of me”, “I have high expectations for the people who are important to me”, respectively). Items are rated on a 7-point scale (1 = *strongly disagree*, 7 = *strongly agree*); higher scores indicate greater perfectionism. Previous studies have shown good internal consistency in nonclinical samples (SOP α = .86; SPP α = .87; [1]), and the current sample had comparable internal consistency (SOP α = .80; SPP α = .87).

#### The Parity Task

The parity task [35], which has been effective in recent effort studies [25,36], served as the mental effort challenge. This task asks participants to decide if the parity of two numbers is the same (i.e., both are even or both are odd) or not (i.e., one number is even and the other is odd). The two numbers are presented on opposite sides of a word (e.g., “8 CHAIR 2”) so the participant must ignore the word in the middle and focus on the two numbers. The word in the middle of the numbers changed between 12 commonplace neutral nouns (e.g., *chair*, *bench*, and *boat*). The numbers used were 2, 3, 5, and 8. Participants were told to press a yellow button if the numbers had the same parity and a blue button if the numbers had different parities. The parity task was delivered using Direct RT (Empirisoft, NY). Responses were collected on a high-speed keyboard with 1 ms accuracy.

The parity task allowed people to work at their own pace: the stimulus remained on the screen until participants responded. As an incentive, people learned that they would receive 3 cents, paid in cash at the end of the experiment, for each correct response. The task lasted for 3 minutes. The task’s goal was to get as many items correct as possible within the 3 minute task period (rather than, for example, to avoid mistakes or to be fast regardless of accuracy), and the incentive of 3 cents was given for correct responses only. The number of correct items is thus our primary measure of task performance.

After completing the parity task, participants completed the following self-report items: “In your opinion, how easy or hard was this task?” (1 = *very easy*, 7 = *very hard*), and “In your opinion, how well did you do on the parity task?” (1 = *very poorly*, 7 = *very well*).

#### Physiological Assessment

The cardiovascular measurements were collected with a Mindware Bionex hardware system (Mindware, Gahanna, OH), which continuously measured electrocardiogram (ECG) and impedance cardiogram (ICG) signals. The ECG was obtained with the modified Lead II configuration with three spot electrodes. The ICG was obtained with four spot electrodes. Two electrodes were used as the receiving electrodes and were placed on the front of the participant’s body (one placed on the left collarbone horizontal to the jugular notch and one placed at the bottom of the sternum). Two other electrodes were used as the sending electrodes and were placed on the participant’s back 1.5 inches higher and lower, respectively, than the upper and lower receiving electrodes. All signals were sampled at 1,000 Hz and were filtered offline (ECG and dZ/dt: .5 to 45 Hz; Z_0_: 10 Hz cutoff; 60 Hz notch filter). Respiration rate (cycles per minute) was derived from the Z_0_ thoracic impedance signal [37,38].

PEP, the time interval in ms between onset of ventricular depolarization and the opening of the aortic valve [39,40], was calculated by the difference between the ECG Q-point [41] and the dZ/dt B-point. Ensemble averages were created by averaging over all of the beats for a 60-second period, using IMP 3.1 (Mindware, Gahanna, OH). The B-point was identified using Lozano’s [42] method, which approximates the B position via regression modeling (i.e., the RB interval is estimated as 55% of the RZ interval, plus 4 ms). A small proportion of beats and ensemble points were corrected as needed.

RSA was computed as high-frequency heart rate variability (in the .15 Hz to .40 Hz range) using the HRV 3.1 software (Mindware, Gahanna, OH). The software automatically identified the R-peaks in the ECG series, and these were visually inspected for errors and artifacts and manually corrected in a small number of cases prior to analysis. For each 60-second period, spectral power values were calculated via a fast Fourier transformation on the interbeat interval series, which had been detrended, centered, and tapered with a Hamming window.

## Results

### Descriptive Statistics and Analytic Approach

Descriptive statistics are presented in Table 1. For each physiological outcome, scores for the 5 baseline minutes were averaged to form a baseline value, and scores for the 3 parity task minutes were averaged to form a task value. We analyzed the data using regression models, as in our recent work [43,44]. Reactivity scores—change from baseline to task—were computed by subtracting the baseline value from the task value. These reactivity scores served as the dependent variables. The predictors were the perfectionism factors (SOP and SPP) and the physiological baseline value, which was included to control for potential initial-value effects. The predictors were centered at the sample’s grand mean, giving them a mean of 0. The intercept thus represents the average within-person change from baseline to task, and the regression coefficients represent how the predictors influence reactivity.

**Table 1:** Descriptive statistics.

	*M*	*SD*
SOP	4.53	0.91
SPP	3.86	0.77
PEP Baseline	121.09	10.48
PEP Task	119.42	11.49
RSA Baseline	5.92	1.18
RSA Task	6.20	1.11
Respiration Baseline	18.92	2.66
Respiration Task	18.76	2.52

**Note.** SOP = self-oriented perfectionism; SPP = socially prescribed perfectionism; PEP = pre-ejection period (ms); RSA = respiratory sinus arrhythmia (in ms^2^); Respiration = respiration rate (cycles per minute). SOP and SPP are averages of items with 1–7 scales.

The models were estimated using Mplus 7.4, using maximum likelihood with robust standard errors. Unless noted otherwise, all regression coefficients are standardized, and 95% confidence intervals are in square brackets. The standardized regression weights are can be viewed as effect sizes in the *r* metric based on conventional cut-offs: .10 (small), .30 (medium), and .50 (large). The raw data and Mplus input are available at Open Science Framework (https://osf.io/8sjhr/).

### Pre-ejection Period (PEP)

Did PEP change in response to the task, and did perfectionism moderate this change? The difference in PEP from the baseline to the task was significantly different from zero: *M* = -1.27 [-2.31, -.22], *SE* = .53, *p* = .017. The negative value indicates that PEP was faster in the task than in the baseline. Faster PEP values reflect greater sympathetic activation [23], so this finding suggests greater sympathetic activation during the task and replicates much past research on PEP as a marker of mental effort [25,36,45].

Perfectionism did not predict PEP reactivity. Neither SOP (β = -.09 [-.27, .09], *p* = 0.323) nor SPP (β = .03 [-.17, .22], *p* = 0.803) significantly predicted PEP reactivity scores. In addition, the baseline PEP values did not relate to PEP reactivity (β = -.07 [-.24, .11], *p* = .466). The model *R*^2^ was small (.01). These results indicate that participants as a group showed increased effort—as evidenced by the significant decline in PEP from baseline to task—but individual differences in perfectionism did not moderate effort.

### Respiratory Sinus Arrhythmia (RSA)

A similar regression model was estimated for RSA reactivity from baseline to task. Respiration rate during the task was included as a predictor to control for potential effects of respiration [46]. RSA reactivity was significant (*M* = .21 [.07, .35], *SE* = .07, *p* = .003), reflecting an increase in RSA from baseline to task. As noted earlier, increased RSA was found in an earlier study with this task [25] and is consistent with Segerstrom’s model of self-regulation and HRV [34,47,48].

Perfectionism, however, did not significantly predict variation in RSA reactivity. Neither SOP (β = -.06 [-.28, .16], *p* = 0.594) nor SPP (β = .05 [-.16, .26], *p* = 0.656) significantly predicted RSA reactivity scores. Only respiration rate (β = -.31 [-.47, -.14], *p* < .001) and baseline RSA (β = -.45 [-.62, -.28], *p* < .001) significantly predicted RSA reactivity. The model *R*^2^ was .25. Thus, RSA values increased for the sample overall during the task, but neither SOP nor SPP significantly predicted RSA change.

### Task Performance and Self-Report Outcomes

Table 2 displays the descriptive statistics and standardized effects for task performance and the subjective self-report items. A multivariate regression model estimated the effects of SOP and SPP on the number of correct items, response times for correct responses, self-reported task difficulty, and self-reported performance. The primary performance outcome was the number of correct items because the task’s goal and incentive structure emphasized getting as many correct as possible.

**Table 2:** Standardized Effects of SOP and SPP on parity task performance and self-report outcomes.

	*M*	*SD*	SOP			SPP		
			β	*SE*	*p*	β	*SE*	*p*
Number Correct	95.31	15.01	.13	.10	0.203	-.16	0.09	0.088
RT	945.88	294.43	-.05	.10	.606	.07	.10	.470
How Hard	3.92	1.68	-0.19	0.11	0.072	0.27	0.10	0.005
How Well	3.68	1.50	0.07	0.11	0.523	-0.10	0.10	0.317

**Note.** The regression coefficients are standardized. RT = response times for correct responses (in ms).

Neither SOP nor SPP significantly predicted the number of correct responses on the parity task; the effect for SPP was marginal (β = -0.16 [-.34, .02], *p* = 0.008; see Table 2), suggesting that people higher in socially prescribed perfectionism tended to do worse. For response times, which were necessarily highly correlated with the number of correct responses (*r* = -.72), neither SOP (β = -0.05 [-.26, .15], *p* = 0.606) nor SPP (β = .07 [-.12, .27], *p* = 0.470) had a significant effect. For self-reported task difficulty, SPP significantly predicted ratings of how hard the task was (β = 0.27 [.08, .46], *p* = 0.005), reflecting higher rated difficulty at higher levels of SPP. SOP’s effect on self-reported task difficulty was marginal but in the other direction (β = -0.19 [-.39, .02], *p* = 0.072): self-oriented perfectionists tended to rate the task as easier. Neither SOP nor SPP significantly predicted participants’ self-report ratings of how well they did on the task (see Table 2).

## Discussion

The current study examined how individual differences in perfectionism are related to effort, measured by autonomic cardiac reactivity during a self-paced task with monetary incentives. The parity task did engage participants as expected. The sample as whole showed faster PEP values during the task, indicating greater sympathetic reactivity, as well as higher RSA values during the task, indicating greater parasympathetic reactivity. The task and its incentives thus successfully motivated the participants in ways that past research would predict. These reactivity effects, however, were not moderated by perfectionism. Neither SOP nor SPP significantly predicted the degree of physiological change from baseline to task for either PEP or RSA. The sample as a whole thus showed increased effort engagement, but differences in how much people engaged were not associated with perfectionism.

The findings from the current study provide additional support for motivational intensity theory and for co-activation of both branches of the autonomic nervous system. Overall, people tried harder during the task, which was evidenced by increased sympathetic and parasympathetic activation. This fits past research, which finds that tasks with unfixed difficulty provide a paradigm where importance of success can be easily measured with effort intensity [19,30]. Few studies have examined both branches of the autonomic nervous system during appetitive tasks requiring effort. However, these findings, along with Silvia and colleagues’ [25] findings, show evidence of a co-activated profile. A review by Segerstrom and colleagues [34] also highlights evidence of a co-activated profile, which may occur during a task that is both challenging and requires self-regulation. Moreover, research indicates that co-activation may be an optimal profile for short-term engagement [33] because it increases cardiac efficiency via higher contractility and a slower heart rate, which affords longer ventricular filling times [49].

The lack of interactions involving perfectionism suggests that perfectionism may not influence effort measured by physiological reactivity. Past research has shown that individual differences in perfectionism predict effort measured by self-reported effort and time spent practicing [12,14,15]. However, within the current study, perfectionism did not predict effort in a paradigm that has been effective in examining whether individual-differences predict effort (i.e., grit, self-focused attention, and anhedonic depression; [25,36,43]). Moving forward with research on perfectionism and motivation, it will be important to use physiological measures of effort in addition to self-report and performance measures because these indicators of effort commonly diverge.

Additionally, perfectionism predicted task performance and appraisal of task difficulty. SPP predicted higher ratings on the self-report item assessing the perception of task difficulty on the parity task. This finding fits with past research, which found that people high on SPP are more likely to rate tasks as more difficult [50]. Furthermore, SPP had a marginally significant, small effect (β = -.16) on the number of correct responses, suggesting they were somewhat less effective in working toward the task’s goal of getting as many correct responses as possible. Maladaptive perfectionism is either negatively or inconsistently associated with performance [6,11,13,14]. The findings from the current study suggest that people high on SPP perceived the task to be more difficult and did not perform as well on it, which contributes to the literature suggesting that SPP may have more negative motivational and performance outcomes.

It is possible that the parity task used in the current study somehow failed to activate perfectionistic goals that would draw out individual differences in effort. Although the parity task isn’t appreciably different from other tasks that have been used to examine perfectionism [14,51,52], it is fundamentally an appetitive task that seeks to activate approach-oriented motivation by offering incentives that are tied to performance and allowing people to work as quickly or slowly as they like. Future studies could use priming techniques to increase the importance of tasks by priming self-relevance or social evaluation [51]. Additionally, SOP was expected to predict effort intensity based on previous findings measuring effort with self-report and performance outcomes; however, the current study used an extrinsic incentive (i.e., 3 cents per correct answer) to increase motivation during the task. SOP has been associated with the pursuit of intrinsically-motivated goals. Therefore, future research should examine whether there is an effect of SOP on effort intensity using an intrinsic motivation task.

In the current study, perfectionists’ appraisal of their performance was not associated with levels of perfectionism. However, performance appraisal was collected with only a single item. Past research on perfectionism and task performance suggests that the different domains of perfectionism may have differing physiological reactions to perceived versus actual task performance [27]. Furthermore, research suggests that adaptive forms of perfectionism are associated with higher levels of self-efficacy [3,6], which may impact one’s motivation during tasks. Therefore, future studies should include more detailed measures of perceived appraisal of performance to examine whether this explains increased or decreased effort in perfectionists. More extensive measurement of perfectionists’ evaluation of tasks, their performance, and their physiological effort may allow for further understanding of the increased likelihood of that perfectionists engage in maladaptive motivational behavior.

Another limitation of the current study is the inclusion of only female participants. This was due to the participant pool being comprised of mostly female students and the limited availability of male research assistants. Based on previous research, gender differences have occasionally been found for physiological reactivity as measured by diastolic blood pressure [53,54] and heart rate [53], although the motivational intensity literature as a whole finds few gender differences.

The current study highlights the importance of assessing physiological measurements of effort, which can provide additional information about task engagement beyond self-report and behavioral measures. Although research suggests individual differences in perfectionism predict performance outcomes, the current study indicates this may not be due to differences in mental effort measured by autonomic reactivity. In the future, identifying discrepancies between performance, evaluation of performance, and effort may be beneficial for understanding perfectionsts’ tendency towards self-defeating behaviors, such as procrastination and self-criticism.
